# Non-infectious complications of peritoneal dialysis in children

**DOI:** 10.1007/s00467-025-06713-5

**Published:** 2025-03-03

**Authors:** Mattia Parolin, Giovanni Ceschia, Nicola Bertazza Partigiani, Edoardo La Porta, Enrico Verrina, Enrico Vidal

**Affiliations:** 1 Present Address: Department of Medicine (DMED), University of Udine, Udine, Italy; 2 Pediatric Nephrology Unit, University Hospital of Padua, Padua, Italy; 3 Nephrology, Dialysis and Transplantation Unit, IRCCS Istituto Giannina Gaslini, Genoa, Italy; 4 Institute of Pediatric Research “Città Della Speranza”, Padua, Italy

**Keywords:** Peritoneal dialysis (PD), Non-infectious complications, Peritoneal membrane failure, Encapsulating peritoneal sclerosis, Catheter dysfunction

## Abstract

Peritoneal dialysis (PD) remains a cornerstone treatment for children with stage 5 chronic kidney disease, offering significant benefits in terms of quality of life and patient autonomy compared to hemodialysis. Recent advances in care protocols, particularly in infection prevention, have led to a substantial reduction in peritonitis and other infectious complications in pediatric patients on PD. Despite these improvements, non-infectious complications continue to pose significant challenges to the long-term efficacy of PD. Mechanical issues, such as catheter malposition and dysfunction, are common and can lead to discomfort and diminished dialysis efficiency. A more serious concern is peritoneal membrane failure, which results from prolonged PD, particularly with glucose-based solutions, and recurrent peritonitis, leading to structural changes in the membrane. Encapsulating peritoneal sclerosis (EPS), although rare, is a severe and often devastating complication that significantly impacts patient morbidity and mortality. Despite its low incidence, EPS underscores the importance of careful monitoring and management of long-term PD patients. Additional complications, including metabolic disturbances, pancreatitis, and hemoperitoneum, further complicate care. Looking ahead, improving catheter management, preserving peritoneal membrane function, and exploring new dialysis solutions are essential to reducing these complications and optimizing outcomes for pediatric patients on PD.

**Graphical abstract:**

**Figure:**
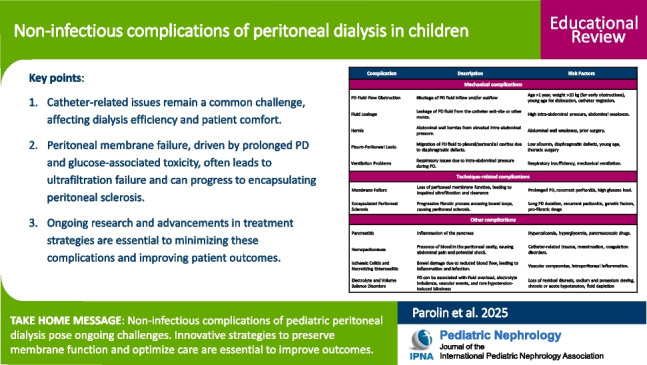
A higher resolution version of the Graphical abstract is available as Supplementary information

**Supplementary Information:**

The online version contains supplementary material available at 10.1007/s00467-025-06713-5.

## Introduction

The Standardizing Care to Improve Outcomes in Pediatric End-Stage Renal Disease (SCOPE) collaborative has made significant strides in enhancing outcomes for children with kidney failure by implementation of standardized care protocols. A key focus of these efforts has been reducing peritonitis, a major complication in pediatric patients undergoing peritoneal dialysis (PD). Through the use of comprehensive care bundles—including optimized catheter insertion techniques, structured training programs for patients and caregivers, and systematic follow-up procedures—the initiative has led to a substantial decline in peritonitis rates. Before its launch in 2011, the average monthly peritonitis rate was 0.53 infections per patient-year, which decreased to 0.30 infections per patient-year 7 years post-implementation [1]. This remarkable improvement highlights the critical role of evidence-based, standardized practices in optimizing PD care and preventing infections.

Beyond infections, non-infectious complications of peritoneal dialysis (NICPD), often mechanical or technique-related, represent a critical challenge in maintaining long-term PD therapy in children. Issues such as catheter malfunction, peritoneal leaks, hernias, and ultrafiltration failure (UFF) are common and can compromise treatment sustainability (Table 1). The prevalence of NICPD tends to increase with prolonged therapy, leading to more frequent occurrence of “late” NICPD. These complications not only impact the efficacy of PD but may also necessitate interventions that disrupt treatment continuity or require a shift to alternative kidney replacement therapies. Addressing these challenges demands a deeper understanding of their mechanisms and the development of targeted prevention and management strategies.

**Table 1:** Summary of non-infectious complications of peritoneal dialysis in children

Complication	Description	Risk factors
**Mechanical complications**		
PD fluid flow obstruction	Blockage of PD fluid inflow and/or outflow	Age > 1 year, weight > 10 kg (for early obstructions), young age for dislocation, catheter migration
Fluid leakage	Leakage of PD fluid from the catheter exit site or other routes	High intra-abdominal pressure, abdominal weakness
Hernia	Abdominal wall hernias from elevated intra-abdominal pressure	Abdominal wall weakness, prior surgery
Pleuro-peritoneal leaks	Migration of PD fluid to pleural/pericardial cavities due to diaphragmatic defects	Low albumin, diaphragmatic defects, young age, thoracic surgery
Ventilation problems	Respiratory issues due to intra-abdominal pressure during PD	Respiratory insufficiency, mechanical ventilation
**Technique-related complications**		
Membrane failure	Loss of peritoneal membrane function, leading to impaired ultrafiltration and clearance	Prolonged PD, recurrent peritonitis, high glucose load
Encapsulating peritoneal sclerosis	Progressive fibrotic process encasing bowel loops, causing peritoneal sclerosis	Long PD duration, recurrent peritonitis, genetic factors, profibrotic drugs
**Other complications**		
Pancreatitis	Inflammation of the pancreas	Hypercalcemia, hyperglycemia, pancreatotoxic drugs
Hemoperitoneum	Presence of blood in the peritoneal cavity, causing abdominal pain and potential shock	Catheter-related trauma, menstruation, coagulation disorders
Ischemic colitis and necrotizing enterocolitis	Bowel damage due to reduced blood flow, leading to inflammation and infection	Vascular compromise, intraperitoneal inflammation
Electrolyte and volume balance disorders	PD can be associated with fluid overload, electrolyte imbalance, vascular events, and rare hypotension-induced blindness	Loss of residual diuresis, sodium and potassium sieving, chronic or acute hypotension, fluid depletion

This review explores the prevalence, clinical manifestations, and outcomes of NICPD in children, emphasizing its effect on patient health and therapy durability. It delves into underlying mechanisms, risk factors, and complications, including peritoneal membrane failure and encapsulating peritoneal sclerosis (EPS). Additionally, it evaluates current strategies for prevention and management, identifies gaps in clinical practice, and proposes improvements in catheter care, membrane preservation, and the use of biocompatible solutions. By addressing these critical challenges, the goal is to optimize long-term outcomes and enhance the quality of life for children on PD.

## Mechanical complications

Mechanical complications in pediatric PD often stem from issues with the peritoneal catheter or increased intra-abdominal pressure caused by PD fluid. Common challenges include fluid flow obstruction, peri-catheter leakage, inflow or outflow discomfort, and hernias. Other mechanical issues, such as hydrothorax, gastroesophageal reflux, and respiratory encumbrance, may also arise. Due to their unique anatomical and physiological characteristics, young infants and newborns are particularly susceptible to these complications [2].

### PD fluid flow obstruction

Outflow failure, defined as incomplete drainage of instilled PD fluid, is typically caused by constipation, catheter migration, intraluminal or extraluminal catheter obstruction, and catheter kinking. Inflow obstruction, which prevents PD fluid from entering the peritoneal cavity, is most commonly due to intraluminal blockage by fibrin or blood, catheter kinking, or displacement of the catheter outside the pelvic cavity. Constipation is one of the most common causes of mechanical complications. Notably, it can result from PD, primarily due to reduced peristalsis caused by increased intra-abdominal pressure, electrolyte imbalances, fluid restrictions, low-fiber diets, and medications. Additionally, uremia and the catheter may further compromise intestinal motility [3]. Large studies report obstruction rates ranging from 5 to 44% [4, 5]. Recent findings identify age over 1 year and weight above 10 kg as significant risk factors for early catheter obstruction [6]. Conversely, other studies suggest that infants are more prone to catheter dislocation and malfunction [7].

Optimal catheter selection, surgical technique, and postoperative care are critical for preventing PD fluid flow obstruction [8]. In pediatric acute PD, soft silicone cuffed catheters, such as the Tenckhoff, have shown clear advantages over non-cuffed Teflon catheters, like the Cook, by significantly reducing complications (9% vs. 49%) and allowing a longer mean duration of dialysis (16.5 vs. 4.9 days) [9]. In line with these findings, guidelines for chronic PD recommend a double-cuff Tenckhoff catheter for children over 3 kg, with the superficial cuff positioned 2–3 cm from the exit site in subcutaneous tissue, as the standard in North America and Europe [5, 10, 11]. However, despite being widely accepted, these recommendations often clash with the unavailability of Tenckhoff catheters in certain regions, particularly in resource-limited settings. In such cases, other strategies are implemented to ensure access to kidney replacement therapy for children, utilizing available resources even if they do not fully align with current guidelines [12].

Regarding catheter configuration, earlier studies recommended a curved intraparietal tunnel (swan neck shape) over a straight one and found no significant difference between straight and curled intraperitoneal catheter configurations [8, 13]. Data from the International Pediatric Peritoneal Dialysis Network (IPPDN) Registry indicate a higher odds ratio of 1.30 (95% CI: 1.03–1.59) for access revision when using catheters with a swan neck tunnel and a curled intraperitoneal segment [5]. Similar findings were reported in a recent multicenter study from Turkey, which noted an increased rate of mechanical complications in presence of a swan neck tunnel [14].

Several studies support omentectomy as an effective method for preventing extraluminal catheter obstruction. A multicenter study from the Pediatric Nephrology Research Consortium (PNRC) involving 184 children on chronic PD found that catheter revision/replacement occurred in 23.9% of those with omentectomy versus 52.2% of those without, with a 63% reduction in revisions after adjusting for covariates [15]. A 2022 systematic review further confirmed effectiveness of omentectomy in reducing peritoneal catheter obstructions [16].

The surgical technique used for PD catheter placement plays a key role in catheter survival. Advanced laparoscopic techniques, which include rectus sheath tunneling, suture fixation, and omentectomy or omentopexy, have shown promising results in both adults and children [17, 18]. While some studies support the benefits of these approaches, others have found no significant differences in success rates between advanced laparoscopic and other methods [19, 20]. Nevertheless, the latest guidelines from the Society of American Gastrointestinal and Endoscopic Surgeons (SAGES) recommend advanced laparoscopic insertion techniques over basic laparoscopic or open methods for both adults and children, even if the panel acknowledges that pediatric data remain scarce and of low quality [21].

A recent SCOPE study analyzed the timing of gastrostomy placement relative to PD catheter placement, showing a trend toward higher infectious risk when performed after the PD catheter, compared to before or simultaneously (*p* = 0.07) [22]. Although not statistically significant, the findings support coordinated planning of gastrostomy and PD catheter placement.

When PD fluid flow is compromised, a systematic diagnostic and therapeutic approach is crucial. Abdominal radiography is typically performed to evaluate catheter position, while ultrasonography (US) offers a complementary tool, showing significant correlation with laparoscopic findings in cases of malfunction [23]. Initial management includes addressing constipation and flushing the catheter with saline to clear intraluminal clots or debris [24]. Persistent obstruction may warrant the use of intraluminal tissue plasminogen activator (tPA), which has shown efficacy in resolving occlusions unresponsive to flushing [25]. For catheters displaced from the rectovesical pouch, guidewire manipulation under fluoroscopic guidance can effectively restore position, with data from the Italian Registry of Pediatric Chronic Dialysis (IRPCD) indicating a 25% success rate [4]. If these measures fail, surgical repositioning or catheter removal is necessary.

### Peritoneal dialysis fluid leakage

Peritoneal dialysis fluid leakage refers to the escape of fluid from the peritoneal cavity through routes other than the catheter lumen. The main site is the catheter exit site, but fluid can also transfer to nearby spaces or tissues. Leaks are classified as early (within 30 days of catheter insertion) or late. Early leaks, more frequent in infants and young children, often present as peri-catheter leaks. Late leaks manifest as subtle signs like swelling, edema, weight gain, or UFF [2, 7].

The incidence of peri-catheter leaks in pediatric studies varies widely, ranging from 3 to 41.5%, reflecting differences in patient populations, study designs, and clinical practices [4, 7, 26]. Key risk factors include elevated intraperitoneal pressure (IPP), often influenced by the filling volume, and abdominal wall weakness, which may be congenital, related to prior abdominal surgery, or due to the patient’s young age. To minimize leakage risk, it is crucial to manage IPP effectively. Since pressure is directly proportional to the instilled volume, initiating PD with low fill volumes has been widely recommended as a preventative strategy [27]. Fischbach et al. introduced a practical method to monitor IPP using a central venous pressure scale connected to the PD tubing system. Their findings suggest that maintaining IPP below 18 cm H_2_O during the filling phase significantly reduces the likelihood of mechanical complications, including leaks, hernia formation, and abdominal discomfort [28].

The timing of PD initiation after catheter insertion significantly impacts the risk of leakage. A retrospective pediatric study found that delayed initiation (> 14 days) was associated with fewer leaks [29], a finding corroborated by recent research showing all leaks occurred with < 14-day break-in periods [26]. Based on this evidence, SAGES guidelines recommend waiting at least 14 days after catheter placement before use [21].

Implantation method and cuff number have been studied as factors related to fluid leakage risk, but no significant correlation was found [7]. However, another study found that a paramedian catheter entry site, compared to midline, showed a trend toward significance, suggesting that insertion site may influence leakage risk [4]. To reduce leakage, some centers have tested fibrin glue applied to the cuff suture, with positive results. In a randomized study of 45 children, leakage occurred in 9% of catheters in the sealant group versus 57% in controls (*p* < 0.05) [30]. Another small study also found fibrin glue effective when used to manage leaks [31]. However, strong evidence supporting its routine use is still lacking.

When leakage is diagnosed, prompt action is essential. Reducing fill volumes can lower IPP, while temporarily switching to hemodialysis (HD) allows tissue consolidation. If conservative measures fail, surgical repair or, more rarely, catheter replacement may be needed [24].

### Hernia

In the presence of abdominal wall weakness, elevated IPP from PD fluid instillation may contribute to hernia formation. Infants and young children are particularly at risk due to physiological anatomical weaknesses in correspondence with the inguinal canals, umbilicus, and linea alba. Prior abdominal surgery or an abdominal stoma further increases this risk. Hernia incidence is reported to be as high as 30% in pediatric populations [4, 5], with infants having a threefold higher risk compared to older children, largely due to the prevalence of a patent processus vaginalis [13].

Hernias often present as painless swelling, though discomfort may occasionally occur, while pain is rare, typically indicating complications. Hernias can also compromise PD effectiveness, as increased dwell volumes may enlarge hernias, reducing dialysis efficiency. These factors suggest considering prophylactic closure of the processus vaginalis during catheter insertion in neonates and young infants starting on chronic dialysis; however, neither European nor North American PD guidelines currently recommend this procedure [10, 11]. In a recent study, Wong et al. found that a pediatric laparoscopic approach—combining catheter placement, omentectomy, and closure of any patent processus vaginalis—significantly reduced the risk of inguinal hernia reoperation (0/33 vs. 5/32, *p* = 0.02) and catheter failure (HR 0.11; 95% CI: 0.04–0.31) [32]. Although promising, current data remain insufficient to advocate for a routine preemptive approach.

### Pleuro-peritoneal and pericardio-peritoneal leak

**Fig. 1:**
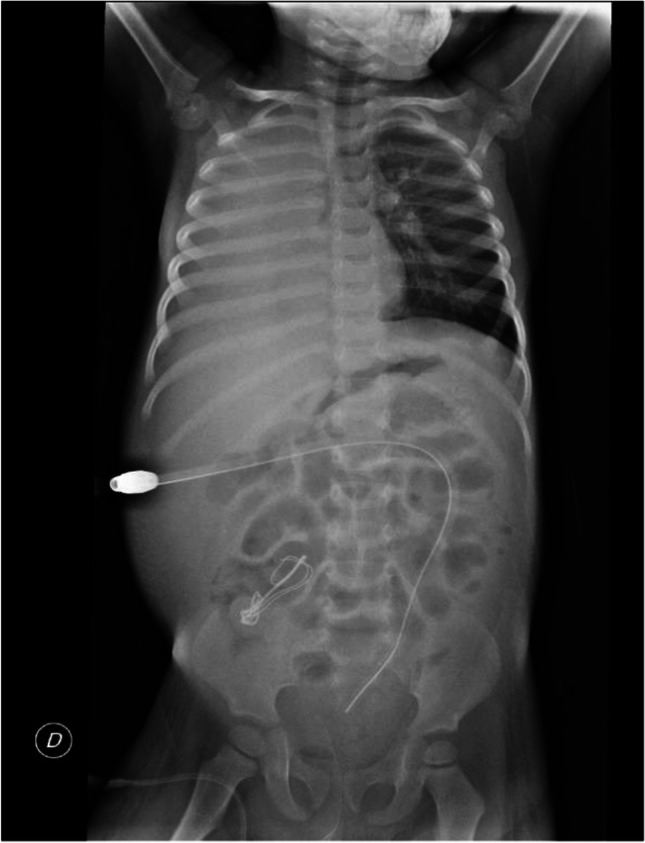
Right-sided pleural effusion in a 2-year-old patient undergoing peritoneal dialysis. The patient experienced respiratory distress, necessitating a temporary switch to extracorporeal kidney replacement therapy and the placement of a chest tube for 2 days

Pleural- and pericardial-peritoneal fistulas (PPF, PcPF) are rare but potentially life-threatening complications of PD. In a survey conducted by the European Pediatric Dialysis Working Group (EPDWG) involving 1506 children on chronic PD, the prevalence was 0.66% [33], aligning with reports in adults [34]. The mechanism is poorly understood but often involves pleuro-peritoneal communication, typically due to congenital or acquired defects in the tendinous portion of the hemidiaphragms, allowing dialysis fluid to migrate from the abdomen. While no direct link exists between kidney disease and PPF, diaphragmatic defects have been associated with *WT1* mutation syndromes [35]. Proposed risk factors include low albumin levels, increased intra-abdominal pressure (e.g., large cystic kidneys in ARPKD), hypotonic musculature in children with trisomy 21, and altered lymphatic drainage [27, 33]. Younger age also appears to be a significant risk factor, with 90% of affected children in the EPDWG survey under 3 years old [33]. The pleural-peritoneal connection is almost exclusively right-sided due to anatomical factors, including the protective role of the heart and pericardium on the left, the prevalence of right-sided diaphragm defects, right colonic peristalsis directing fluid upward, and liver movement enhancing fluid translocation (Fig. 1).

Clinically, PPF may be asymptomatic with minimal pleural fluid, or present with respiratory symptoms such as polypnea or severe ventilatory impairment. It can also exacerbate ultrafiltration (UF) issues, potentially necessitating the use of hyperosmolar dialysis solutions, which may further increase intra-abdominal pressure and worsen hydrothorax. Prompt recognition requires a high index of suspicion. Respiratory symptoms in PD patients should prompt chest radiography and US to detect hydrothorax. Diagnostic thoracocentesis reveals transudative effusion with high glucose levels (> 300–400 mg/dL or a pleural fluid-serum glucose gradient > 50 mg/dL). When anatomical confirmation is needed, imaging modalities like MRI or CT are preferred, though methylene blue infusion into the peritoneal cavity can be diagnostic but carries a risk of chemical peritonitis [33].

Management primarily involves temporary discontinuation of PD for 2–6 weeks to allow diaphragmatic defect resolution [36]. In symptomatic cases, thoracocentesis may be required. Refractory cases may necessitate interventions such as pleurodesis or surgical repair. In the EPDWG cohort, all affected patients discontinued PD, but only 3 out of 10 attempted resumptions, leaving unclear the likelihood of successfully restarting PD after such complication [33].

## Technique-related complications

### Membrane failure

Chronic exposure to PD solutions, particularly glucose-based fluids, is the primary driver of membrane transformation [37]. Bartosova et al. demonstrated that peritoneal membrane alterations occur early in PD and are predominantly influenced by dialysis vintage and cumulative glucose exposure, with peritonitis playing a lesser role in structural changes [38]. Progressive damage results in increased small solute transfer, UFF, and, in severe cases, EPS. These alterations can begin within months of PD initiation, as advanced glycation end-products (AGEs) induce mesothelial-to-mesenchymal transition, leading to interstitial accumulation of cytokeratin-positive fibroblasts, secretion of vascular endothelial growth factor, chronic inflammatory peritoneal vasculopathy, neoangiogenesis, and ultimately fibrosis [39]. These changes cause hyperpermeability and loss of the crystalloid osmotic gradient, significantly increasing the risk of membrane failure, which often becomes evident after 2 years of PD [37].

Although recurrent peritonitis has been historically associated with peritoneal deterioration, evidence indicates that peritonitis episodes alone do not drive peritoneal transformation in the early years of PD [38]. However, they still contribute to membrane dysfunction by releasing cytokines such as TGF-beta, IL-1beta, IL-6, and TNF-alpha, promoting angiogenesis and fibrosis [40]. Additionally, genetic polymorphisms influencing cytokine expression and endothelial nitric oxide synthase activity can exacerbate neoangiogenesis [41]. Schaefer et al. demonstrated that even neutral pH and low-GDP dialysis fluids can induce early peritoneal membrane alterations in pediatric PD patients [42]. Their study found increased submesothelial thickening, angiogenesis, and early signs of vasculopathy, suggesting that despite reduced GDP exposure, these solutions still contribute to structural peritoneal changes.

The aging of the peritoneal membrane makes UFF directly proportional to PD duration, affecting approximately 20% of patients after 2 years and 35% after 4 years of treatment [43]. This complication significantly contributes to PD dropout, often leading to fluid overload and cardiovascular complications, particularly in anuric patients [44]. The Peritoneal Equilibration Test (PET) is the most widely used diagnostic tool to evaluate peritoneal membrane function and determine UFF causes. In long-term PD patients with UFF, a high transport status suggests rapid loss of the osmotic gradient, indicative of type 1 UFF, whereas a low transport status may reflect impaired glucose transport, as seen in type 2 UFF [43].

Hypertonic solutions and short dwell times are typically prescribed to preserve UF in cases of membrane failure. Icodextrin, a large water-soluble glucose polymer derived from corn starch, can achieve slow and sustained UF with a safe profile, even in children [45]. However, its use is limited by its slow kinetics and systemic absorption, restricting it to a single prolonged daily exchange. Despite this limitation, it reduces the glucose load by approximately 30% [46]. Amino acids–based solutions offer an alternative but share similar limitations, as only one exchange can be used per day due to the risk of acidosis and increased urea levels [47].

To improve peritoneal biocompatibility and mitigate glucose-associated toxicity, novel solutions containing osmotic agents such as taurine, polyglycerol, carnitine, and xylitol have been explored preclinically, aiming to reduce peritoneal exposure to glucose while maintaining adequate UF [48]. Xylitol has emerged as a promising candidate due to its favorable metabolic profiles and potential to improve peritoneal transport efficiency, with studies suggesting that it may help reduce the inflammatory and fibrotic responses associated with glucose-based PD fluids [49], though its long-term safety requires careful evaluation. Additionally, strategies have been investigated to incorporate protective agents into PD solutions to counteract glucose-induced toxicity by antagonizing the pro-inflammatory and fibrotic pathways triggered by glucose metabolism [50].

### Encapsulating peritoneal sclerosis

EPS is a rare but extremely serious complication of long-term PD. It is characterized by a progressive, multifactorial fibrotic, and inflammatory process that leads to generalized peritoneal sclerosis, encasing bowel loops and impairing intestinal function and motility. Time on PD and oxidative stress to the peritoneal membrane are key contributors to this fibrotic degeneration. However, not all patients with UFF progress to EPS, suggesting additional factors in a “double hit” model. In this hypothesis, precipitating factors such as severe or recurrent peritonitis (commonly caused by *S. aureus*, *Pseudomonas*, or fungi), certain drugs, discontinuation of PD, and genetic predisposition act on the background of prolonged exposure to PD solutions [51]. A survey by the EPDWG reported a 1.5% prevalence of EPS in children on PD, with a mortality rate of 13.6% (3 out of 22 patients) [52]. In contrast, a retrospective review of the IRPCD noted a higher mortality rate of 43%, similar to rates reported in adult patients (30–56%) [53]. Importantly, EPS can also occur after PD discontinuation, transitioning to HD or kidney transplantation. In these cases, EPS often presents acutely and progresses rapidly [53]. Post-transplant factors, such as the use of calcineurin inhibitors and the cessation of PD-related removal of pro-inflammatory and profibrogenic cytokines, may accelerate EPS development [54].

**Fig. 2:**
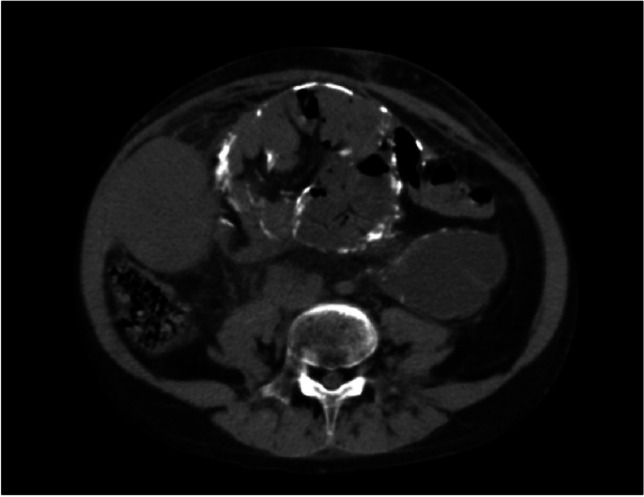
CT scan of a pediatric transplant recipient with encapsulating peritoneal sclerosis, previously treated with peritoneal dialysis, showing peritoneal calcifications

Diagnosing EPS requires clinical suspicion, particularly in patients with long-term PD, membrane failure, and persistent or intermittent signs of intestinal obstruction. Confirmation is typically achieved through a CT scan, which may reveal peritoneal tethering, wall thickening, calcifications, or bowel dilatation (Fig. 2) [55]. Efforts to identify risk factors or early diagnostic tools include cytokine assays, routine CT screening, and peritoneal biopsies [51].

Therapeutic approaches vary based on the stage of EPS. In the early inflammatory stages, discontinuation of PD and medical therapies such as steroids, immunosuppressants (e.g., mycophenolate mofetil, azathioprine, sirolimus), or TGF-beta inhibitors may be beneficial. While no randomized controlled trials exist, steroid therapy is currently used and has shown some efficacy in early disease [51]. Total parenteral nutrition may be necessary to address malnutrition, and surgical intervention is mandatory in cases of bowel obstruction. A debated aspect of managing PD-related complications is deciding whether and when to preemptively switch from PD to HD. Transitioning to HD could potentially prevent UFF-related complications and improve outcomes when PD-related morbidity increases [56]. However, this approach may not necessarily prevent EPS development, given its low prevalence in the PD population and its occurrence even after PD discontinuation. Thus, individualized risk assessment remains crucial in determining the optimal timing and modality for dialysis transitions.

Histological evaluation of peritoneal tissue in children who have discontinued PD reveals a complex interplay of changes [57]. While PD discontinuation is associated with the resolution of PD-induced hypervascularization and a partial reduction in peritoneal inflammation, persistent profibrotic activity remains a concern. This is evidenced by diffuse podoplanin positivity and an absence of mesothelial cell recovery. Notably, this profibrotic activity can persist even after kidney transplantation, potentially exacerbated by the use of calcineurin inhibitors. These findings provide insight into the mechanisms underlying the development of EPS following both PD cessation and kidney transplantation, emphasizing the need for vigilant monitoring and targeted therapeutic strategies in these patients.

### Metabolic effects of glucose absorption

Glucose homeostasis is significantly disrupted in chronic kidney disease (CKD), with children undergoing PD particularly affected by the metabolic impact of glucose-based dialysis solutions. These solutions can exacerbate hyperglycemia, hypertriglyceridemia, and the loss of albumin and high-density lipoproteins (HDL) into the peritoneal cavity. This metabolic burden increases the risk of developing metabolic syndrome, characterized by elevated levels of total cholesterol, triglycerides, low-density lipoprotein (LDL) cholesterol, apolipoprotein A, and lipoprotein(a), alongside reduced HDL levels [58, 59]. Glucose absorption from PD contributes approximately 7–10 kcal/kg per day to the total daily energy intake, accounting for 10–15% of estimated daily energy requirement in children on PD, However, this glucose absorption can negatively impact nutritional status by reducing appetite through its role in satiety regulation. Glucose activates a sensing system in the portal vein, which signals the hypothalamus to suppress exogenous glucose intake, potentially leading to poor feeding. When combined with CKD-related factors such as metabolic acidosis, impaired anabolic hormone response, chronic inflammation, and increased metabolic rates, this effect can exacerbate protein-energy wasting syndrome, a critical issue in pediatric patients on PD [60].

While chronic PD has been associated with a higher initial risk of atherogenesis compared to HD, studies have shown that lipid profiles tend to stabilize or improve with prolonged use. As a result, over time, the lipid profile in pediatric patients on long-term PD becomes comparable to that of those on HD [61].

The metabolic disturbances induced by glucose-based dialysis solutions—such as dyslipidemia, inflammation, and insulin resistance—not only contribute to atherosclerosis but also predispose pediatric patients to long-term cardiovascular morbidity. Notably, cardiovascular disease remains the leading cause of mortality among children undergoing kidney replacement therapy, with approximately one-third of deaths in pediatric PD patients attributed to cardiovascular complications [62]. Alarmingly, mortality rates in children on dialysis are at least 30 times higher than those in the general pediatric population, underscoring the urgent need for targeted strategies to mitigate these risks [63].

## Other complications

### Pancreatitis

In children undergoing chronic PD, acute pancreatitis (AP) represents a severe complication, with an incidence of 4 to 50 episodes per 1000 patient-years. According to data from the IRPCD, children on chronic PD face a markedly increased relative risk of AP compared to their peers in the general pediatric population, with a relative risk of 41.3 (95% CI: 1.35–60.5) [64].

The pathophysiology of AP in this population is complex and multifactorial. Vidal et al. proposed the “Kidney-Pancreatic Syndrome,” where impaired kidney clearance of gastrointestinal hormones leads to excessive pancreatic enzyme secretion, contributing to pancreatic dysfunction [64]. Metabolic disturbances, including electrolyte imbalances, uremia, and hypercalcemia—particularly in children with adynamic bone disease—further increase AP risk by inducing acinar cell injury and enzyme activation. The role of PD remains debated, with potential contributions from dialysate composition, acidity, hypertonicity, and calcium content, which may irritate the pancreas or create localized hypercalcemia. Additionally, supraphysiological glucose concentrations in PD fluids can exacerbate hyperglycemia and hypertriglyceridemia, both recognized AP risk factors [58]. Exposure to pancreatotoxic medications, such as ACE inhibitors, may also contribute by causing pancreatic duct obstruction and inflammation.

The diagnosis of AP in patients undergoing PD is based on the revised Atlanta classification, requiring characteristic abdominal pain, elevated serum lipase or amylase levels, and diagnostic imaging findings on CT scan, MRI, or US [65]. In children on PD, epigastric pain is the most common symptom, though infants and toddlers often present with abdominal distension and fever. Diagnosing AP in chronic PD patients is challenging due to symptom overlap with peritonitis; peritoneal fluid amylase levels above 100 U/L can aid in differentiation. Imaging is crucial for diagnosis and assessing complications, with CT and MRI preferred for severe cases [64]. In later stages, cholangio-MRI is favored in children for its lower radiation exposure and effectiveness in detecting secondary strictures.

Supportive care is the cornerstone of AP management in children and includes discontinuing enteral feeding, providing parenteral nutrition, administering antibiotics, and using analgesics [66]. The decision to continue PD is case-dependent, with evidence suggesting that peritoneal lavage may be beneficial in severe cases [67]. However, 42.5% of PD patients with AP require temporary HD due to severe pain or UFF, often linked to paralytic ileus [64]. Outcomes depend on disease severity, timely diagnosis, and appropriate treatment. In a cohort of 12 children with AP from the IRPCD, the mortality rate was 25%, though deaths were not directly caused by AP [64].

### Hemoperitoneum

Hemoperitoneum occurs in 1.2–7% of children on chronic PD [4]. Even a small amount of blood, such as 1 mL in 2 L of effluent, can visibly tint the dialysate. Common causes include catheter-related trauma, which typically results in mild bleeding during PD catheter insertion, manipulation, or removal (< 5% of cases) [14]. However, severe bleeding from vessel or splenic lacerations may require surgical intervention and treatment for hemorrhagic shock. Menstrual bleeding is another frequent cause, affecting over 50% of menstruating patients on PD. This benign hemoperitoneum, often related to ovulation, retrograde menstruation, or endometriosis, generally requires no treatment but should be considered in post-menarche girls [68].

Intra-abdominal diseases can also lead to hemoperitoneum, though these causes are rare. They include splenic or liver rupture, retroperitoneal or iliopsoas hematomas, ruptured ovarian cysts, ectopic pregnancies, or cyst ruptures in autosomal-dominant polycystic kidney disease (ADPKD) [4]. Coagulation disorders, either spontaneous or related to anticoagulant use, may contribute as well, requiring management of the underlying coagulopathy or medication adjustments [69]. Other potential causes include EPS and peritoneal calcifications, which can cause bleeding due to peritoneal inflammation or impaired solute clearance.

Symptoms range from mild discomfort to severe acute abdominal pain, potentially accompanied by signs of peritonitis or hemodynamic instability. Alarm features such as fever, hypotension, severe pain, or a history of trauma warrant immediate attention. Diagnosis involves analyzing peritoneal fluid for cell count, culture, and amylase levels. Significant bleeding, indicated by hematocrit levels in dialysate > 2%, calls for further imaging with US or CT scan [69].

Treatment varies depending on severity. Mild to moderate bleeding often resolves spontaneously, while severe cases may require surgical intervention. Supportive measures include heparin instillation (500 IU/L) to prevent intraperitoneal clotting without systemic effects, rapid dialysate exchanges with room-temperature solutions to promote vasoconstriction, and medication adjustments such as oral contraceptives for menstruating women [69]. In severe or refractory cases, a temporary switch to HD may be necessary. Notably, recurrent hemoperitoneum has not been associated with long-term complications such as UFF or an increased risk of peritonitis [70].

### Ischemic colitis and necrotizing enterocolitis

Ischemic colitis and necrotizing enterocolitis, though rare, are serious gastrointestinal complications in PD patients [71]. These conditions arise from reduced mesenteric and bowel blood flow, often linked to intraperitoneal inflammation, vascular compromise, or pre-existing vascular disease. Clinical manifestations typically include abdominal pain, bloody stools, and systemic signs of infection. Prompt diagnosis and management are essential to prevent progression to bowel infarction and sepsis. Key interventions include discontinuing PD and initiating appropriate surgical treatment when necessary.

### Electrolyte and volume balance disorders

Volume status and electrolyte alterations are common in PD patients, arising from a variety of underlying causes. These disturbances warrant detailed discussion due to their complex pathophysiology, clinical consequences, and significant impact on both short- and long-term outcomes [72]. Maintaining volume homeostasis is a cornerstone of PD treatment, as fluid overload is well recognized for its association with an increased incidence of vascular events and higher mortality risk. Despite its frequent occurrence, managing volume overload remains a diagnostic and therapeutic challenge for pediatric nephrologists. It can result from peritoneal membrane failure or a loss of residual diuresis. While infants with residual kidney function may require additional sodium and water supply, older children often need dietary sodium and fluid restrictions, which can be difficult to implement in daily life. Therefore, PD-associated water and sodium removal plays a key role in maintaining euvolemia and proper sodium balance.

Electrolyte imbalances are also frequently encountered in pediatric PD patients. For instance, hyponatremia is often linked to volume overload; however, in infants, it may result from a mismatch between sodium losses via UF and low sodium intake from infant formulas. In such cases, correction can be achieved through oral or peritoneal sodium supplementation [73]. Both hypokalemia and hyponatremia have been associated with an increased risk of long-term mortality. Notably, hypokalemia has also been linked to a higher risk of peritonitis, likely due to reduced intestinal motility and constipation associated with potassium depletion [74]. Treatment with oral potassium supplementation, targeting a serum concentration of 4–5 mEq/L, has been shown to be both safe and effective in reducing the risk of peritonitis [75].

While less common, volume contraction and hypotension can lead to severe complications, including anterior ischemic optic neuropathy (AION). AION results from infarction of the anterior portion of the optic nerve and is more frequently observed in patients experiencing chronic or acute hypotension, often triggered by bleeding or diarrhea. In pediatric PD patients, this rare but dramatic complication has been associated with irreversible bilateral blindness [76].

## Conclusions

Non-infectious complications of PD in children remain a significant clinical challenge despite advances in care protocols that have substantially reduced infectious complications. Mechanical issues, such as catheter dysfunction, fluid leakage, and hernias, are common and can impact dialysis efficiency and patient comfort. Prolonged exposure to PD solutions and recurrent peritonitis contribute to progressive peritoneal membrane failure, leading to UFF, volume and electrolyte imbalance, and, in severe cases, EPS, which significantly affects morbidity and mortality.

Although rare, complications such as AP, hemoperitoneum, and ischemic colitis further underscore the complexity of managing pediatric PD patients. Early recognition and tailored interventions are critical to improving outcomes.

To optimize long-term care, future strategies should focus on enhancing catheter management techniques, preserving peritoneal membrane function, and developing biocompatible and glucose-sparing dialysis solutions. These approaches will be essential to minimize complications, improve quality of life, and extend the efficacy of PD in the pediatric population.

## Key summary points

1. Catheter-related issues remain a common challenge, affecting dialysis efficiency and patient comfort.
2. Peritoneal membrane failure, driven by prolonged PD and glucose-associated toxicity, often leads to ultrafiltration failure and can progress to encapsulating peritoneal sclerosis.
3. Ongoing research and advancements in treatment strategies are essential to minimizing these complications and improving patient outcomes.

## Multiple choice questions

Answers are given following the reference list.

1. Which of the following is true about Encapsulating Peritoneal Sclerosis (EPS)?

- EPS only occurs during peritoneal dialysis (PD)
- EPS can develop even after PD is discontinued
- EPS is unrelated to the duration of PD treatment
- EPS is effectively treated with randomized steroid therapy trials.

2) Which mechanical complication commonly affects pediatric patients on peritoneal dialysis?

- Catheter malposition and dysfunction
- Hyperglycemia
- Bowel obstruction
- Encapsulating peritoneal sclerosis

3) What is a key factor contributing to the increased risk of pancreatitis in pediatric patients on peritoneal dialysis?

- Elevated blood pressure
- Hypercalcemia and metabolic derangements
- Chronic dehydration
- Excessive dialysis fluid volume

## Supplementary Information

Below is the link to the electronic supplementary material.

Graphical abstract (PPTX 81 KB)
