# Supplementary material for: Non-infectious complications of peritoneal dialysis in children
Source: Pediatr Nephrol. 2025 Mar 3;40(10):3055–66. doi: 10.1007/s00467-025-06713-5 (PMC12402023; doi:10.1007/s00467-025-06713-5)
Supplement: Supplementary file 1 — Graphical abstract (PPTX 81 KB) [file 467_2025_6713_MOESM1_ESM.pptx]

## Slide 1
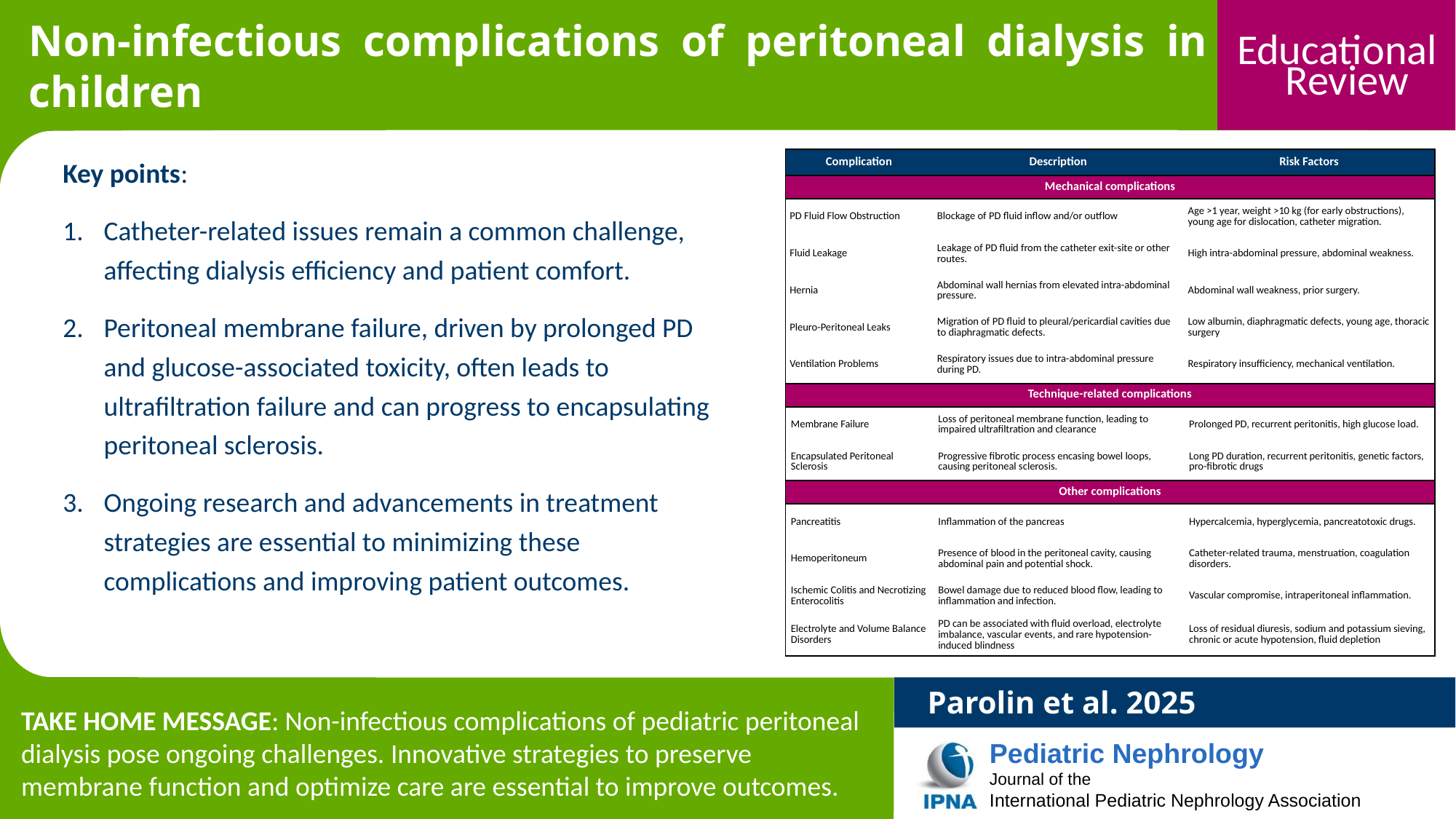

Non-infectious complications of peritoneal dialysis in children
| Complication | Description | Risk Factors |
| --- | --- | --- |
| Mechanical complications | | |
| PD Fluid Flow Obstruction | Blockage of PD fluid inflow and/or outflow | Age >1 year, weight >10 kg (for early obstructions), young age for dislocation, catheter migration. |
| Fluid Leakage | Leakage of PD fluid from the catheter exit-site or other routes. | High intra-abdominal pressure, abdominal weakness. |
| Hernia | Abdominal wall hernias from elevated intra-abdominal pressure. | Abdominal wall weakness, prior surgery. |
| Pleuro-Peritoneal Leaks | Migration of PD fluid to pleural/pericardial cavities due to diaphragmatic defects. | Low albumin, diaphragmatic defects, young age, thoracic surgery |
| Ventilation Problems | Respiratory issues due to intra-abdominal pressure during PD. | Respiratory insufficiency, mechanical ventilation. |
| Technique-related complications | | |
| Membrane Failure | Loss of peritoneal membrane function, leading to impaired ultrafiltration and clearance | Prolonged PD, recurrent peritonitis, high glucose load. |
| Encapsulated Peritoneal Sclerosis | Progressive fibrotic process encasing bowel loops, causing peritoneal sclerosis. | Long PD duration, recurrent peritonitis, genetic factors, pro-fibrotic drugs |
| Other complications | | |
| Pancreatitis | Inflammation of the pancreas | Hypercalcemia, hyperglycemia, pancreatotoxic drugs. |
| Hemoperitoneum | Presence of blood in the peritoneal cavity, causing abdominal pain and potential shock. | Catheter-related trauma, menstruation, coagulation disorders. |
| Ischemic Colitis and Necrotizing Enterocolitis | Bowel damage due to reduced blood flow, leading to inflammation and infection. | Vascular compromise, intraperitoneal inflammation. |
| Electrolyte and Volume Balance Disorders | PD can be associated with fluid overload, electrolyte imbalance, vascular events, and rare hypotension-induced blindness | Loss of residual diuresis, sodium and potassium sieving, chronic or acute hypotension, fluid depletion |
Key points:
Catheter-related issues remain a common challenge, affecting dialysis efficiency and patient comfort.
Peritoneal membrane failure, driven by prolonged PD and glucose-associated toxicity, often leads to ultrafiltration failure and can progress to encapsulating peritoneal sclerosis.
Ongoing research and advancements in treatment strategies are essential to minimizing these complications and improving patient outcomes.
Parolin et al. 2025
TAKE HOME MESSAGE: Non-infectious complications of pediatric peritoneal dialysis pose ongoing challenges. Innovative strategies to preserve membrane function and optimize care are essential to improve outcomes.
